# P57 - Prognostic factors for asthma at school age in infants with atopic dermatitis

**DOI:** 10.1186/2045-7022-4-S1-P112

**Published:** 2014-02-28

**Authors:** Sophia Tsabouri, Manthoula Valari, Konstantinos Douros, Vasiliki Gemou-Engesaeth, Maria-Alexandra Magiakou, Evangelia Papadavid, Maria Theodoridou, Konstantinos Priftis

**Affiliations:** 1 Department of Pediatrics, Child Health Department, University of Ioannina, Greece; 2 2nd Pediatric Clinic, University of Athens School of Medicine, Children’s Hospital “P. and A. Kyriakou”, Athens, Greece; 3 1st Pediatric Clinic, University of Athens School of Medicine, Children’s Hospital “Aghia Sofia”, Athens, Greece; 4 Department of Pediatric Pulmonology and Allergology, 3rd Pediatric Clinic, University of Athens School of Medicine, ”Attikon” General Hospital, Athens, Greece

## Introduction

The prevalence of atopic dermatitis (AD), one of the most common skin disorders seen in infants and children, is increasing, similar to that of other atopic disorders, particularly asthma. Although children showing more severe dermatitis have a higher risk of having more persistent AD, the role of severity as prognostic determinants for childhood asthma is not clearly determined.

## Aim

To determine clinical and laboratory prognostic factors in infants with AD for subsequent development of asthma at school age.

## Materials and methods

89 infants with AD aged 3 to 24 months were recruited and followed up until the age of 8 years. The severity of AD at the time of the initial visit was calculated by using the SCORAD index, whereas the laboratory parameters determined were: peripheral blood eosinophil count, serum ECP, total IgE and specific IgE levels for a panel of food and inhaled allergens. Every second year, parents were interviewed about symptoms and diagnosis relevant to asthma by using a standardized questionnaire. At the end-visit, specific IgEs for the same panel of food and inhaled allergens were measured and, pre- and post-bronchodilator spirometry was performed.

## Statistical analysis

For the purpose of univariate, descriptive analysis Fisher’s exact tests were used and for the multivariate analysis of data logistic regression models.

## Results

The study population the age of 8 consisted of 72 children. They had significantly increased the risk of asthma at school age when sensitized to inhaled allergens in infancy (p<0.001). Asthma development was significantly related with ECP levels (p=0.019, OR: 1.10, CI: 1.01 – 1.20). None of the other analyzed early life factors (ie, severity, sex, presence of other atopic conditions, and family history of atopy) showed any association with subsequent development of asthma.

## Conclusion

AD severity in infancy does not have any effect on the future development of asthma. Prognostic determinants were found to be allergic sensitisation to inhaled allergens, as expected, and ECP levels, probably, as allergic eosinophilic inflammation indicator.

